# A rare disease patient-reported outcome measure: revision and validation of the German version of the Systemic Sclerosis Quality of Life Questionnaire (SScQoL) using the Rasch model

**DOI:** 10.1186/s13023-021-01944-9

**Published:** 2021-08-09

**Authors:** Agnes Kocher, Mwidimi Ndosi, Kris Denhaerynck, Michael Simon, Andrew A. Dwyer, Oliver Distler, Kirsten Hoeper, Patrizia Künzler-Heule, Anthony C. Redmond, Peter M. Villiger, Ulrich A. Walker, Dunja Nicca

**Affiliations:** 1 Institute of Nursing Science (INS), Department Public Health (DPH), Faculty of Medicine, University of Basel, Basel, Switzerland; 2 Department of Rheumatology, Immunology and Allergology, Inselspital, Bern University Hospital, University of Bern, Bern, Switzerland; 3 School of Health and Social Wellbeing, University of the West of England, Bristol, UK; 4 Nursing Research Unit, Inselspital, Bern University Hospital, University of Bern, Bern, Switzerland; 5 Boston College, Connell School of Nursing, Chestnut Hill, MA USA; 6 Department of Rheumatology, University Hospital Zurich, University of Zurich, Zurich, Switzerland; 7 Hannover Medical School, Department, Rheumatology and Immunology, Hannover, Germany; 8 Department of Gastroenterology/Hepatology and Department of Nursing, Cantonal Hospital St. Gallen, St. Gallen, Switzerland; 9 Leeds Institute of Rheumatic and Musculoskeletal Medicine, University of Leeds, Leeds, UK; 10 NIHR Leeds Biomedical Research Centre, Leeds, UK; 11 Department of Rheumatology, University Hospital Basel, Basel, Switzerland; 12 Department of Global and Public Health, Institute for Epidemiology, Biostatistics and Prevention, University of Zurich, Zurich, Switzerland

**Keywords:** Health-related quality of life, Item response theory, Methodology, Patient-centered care, Patient reported outcome measures, Rare diseases, Rasch analysis, Rheumatology, Scleroderma, Systemic sclerosis, Validation study

## Abstract

**Background:**

Rare disease patient-reported outcome measures (PROMs) require linguistic adaptation to overcome the challenge of geographically dispersed patient populations. Importantly, PROMs such as health-related quality of life (HRQoL) should accurately capture responses to patient-identified concerns. The Systemic Sclerosis Quality of Life Questionnaire (SScQoL) is a 29-item tool validated in six languages. Previous evaluation of the German version revealed problems with dichotomous responses. This study aimed to revise the German SScQoL, extend the response structure, and evaluate content and construct validity, reliability and unidimensionality.

**Methods:**

The instrument validation study involved revising the German SScQoL response structure, cognitive debriefing with patients and validation using Rasch analysis. The revised SScQoL was completed by Swiss-German-speaking patients with SSc within the Swiss *MANagement Of Systemic Sclerosis (MANOSS)* study. Rasch analysis was employed to test the validity, reliability and unidimensionality of the revised instrument.

**Results:**

Based on cognitive debriefing with patients (n = 6) dichotomous items were extended to a polytomous 4-point response structure. A total of 78 patients completed the revised SScQoL. Initial analysis of the 29 items suggested the scale lacked fit to the model (χ^2^ = 51.224, *df* = 29, *p* = 0.007). Grouping items into five domains resulted in an adequate fit to the Rasch model (χ^2^ = 5.343, *df* = 5, *p* = 0.376) and unidimensionality (proportion of significant independent *t* tests: 0.045, 95% CI 0.016–0.114). Overall, the scale was well targeted, had high internal consistency (Person Separation Index, PSI = 0.931) and worked consistently in patients with different demographic and clinical characteristics.

**Conclusions:**

The revised German SScQoL has a 4-point response structure and is a valid, reliable measure. Rasch analysis is useful for validating continuous response structure of quality of life measures. Further evaluation of measurement equivalence with other German-speaking cultures is required for multinational comparisons and data pooling.

**Supplementary Information:**

The online version contains supplementary material available at 10.1186/s13023-021-01944-9.

## Background

For many rare diseases, the natural history of the condition is poorly understood especially as it relates to the impact on health-related quality of life (HRQoL). Importantly, patients affected by rare diseases are geographically dispersed. Therefore, validated patient-reported outcome measures (PROMs) including HRQoL are needed in multiple languages. Systemic sclerosis (SSc) is a rare multisystemic, connective tissue disease associated with significant morbidity, physical and psychosocial impact [1]. Pathogenesis is dominated by vascular problems such as vasospasm of digital arteries (Raynaud's phenomenon); inflammation and activation of (auto)immune response; and fibrosis of the skin and visceral organs causing irreversible scarring and organ failure. The disease is heterogeneous in clinical manifestations (e.g. autoantibody profile, disease progression, skin involvement) and patients are typically grouped into two disease subsets: limited cutaneous systemic sclerosis (lcSSc) and diffuse cutaneous systemic sclerosis (dcSSc).

Importantly, SSc is a long-term condition and both disease subsets exhibit multiple symptoms including fatigue, hand stiffness, digital ulcers, shortness of breath, pain, and mouth-, dental- and gastrointestinal-problems [2, 3]. Psychosocial problems such as work disability, depression, fear of disease progression, and body image dissatisfaction are often evident [4, 5]. Accordingly, patients’ quality of life is often severely affected [6, 7]. Notably, the diffuse form (dcSSc) is associated with greater negative impact on quality of life compared to limited SSc (lcSSc) without organ damage [6].

To systematically address the range of SSc effects, it is important to assess disease-specific aspects of HRQoL using an outcome measure with demonstrated reliability and validity for some specific languages. HRQoL measures are fundamental in developing PROMs for chronic conditions to evaluate targeted interventions, increase well-being (e.g., detect need for supportive care), and reduce costs (e.g., earlier detection of relapses) [8–10]. Indeed, to achieve adequate sample sizes, rare disease research relies on registries (e.g. EUSTAR and EUSHNet) and demands international/multicenter collaboration given the limited number of affected individuals [11].

While the Health Assessment Questionnaire (HAQ) is a valid measure of physical disability, and commonly used for evaluating patients, it does not adequately take into account the psychosocial aspects or other disease-specific impact in people with SSc [12]. The Systemic Sclerosis Quality of Life Questionnaire (SScQoL) is the first PROM assessing disease-specific HRQoL in people with SSc [13, 14]. Reay et al. developed the instrument through a multi-phased process comprising qualitative interviews (one-to-one interview and focus groups) with people with SSc; development of the descriptive framework of SSc QoL; development of draft items derived from patients statements (90 items); Rasch analysis and item reduction (researchers with patient input—29 items); test–retest with hypothesis testing and structural equation modelling [14]. The developed SScQoL has 29 items with dichotomous (true/not true) responses, scored as ‘True’ = 1 or ‘Not true’ = 0, total score ranges between 0 and 29 with higher scores indicating a greater impact of the disease and consequently, decreased HRQoL [13, 14]. The items have been grouped into five domains which map onto the International Classification of Functioning, Disability and Health (ICF) framework [13], with scores for each domain ranging as follows: function: 0–6; emotional: 0–13; sleep: 0–2; social: 0–6; and pain: 0–2.

The SScQoL underwent a cross-cultural adaptation according to a five-step procedure described by Beaton et al. and validation in six European countries [13, 15]. As part of the cross-cultural adaptation the translated versions of the SScQoL were first completed by a group of 30 patients in each of the six countries (Germany, France, Italy, Poland, Spain, Sweden, and UK) who commented on the translated version before different versions were sent for psychometric testing using Rasch analysis [13]. Findings of the adaptation suggested a seamless adaptation across all countries but Germany where patients documented problems with 10 items [13]. Specifically, problems were identified in relation to the dichotomous ‘true/not true’ response structure in those items. German patients indicated a desire for a broader response structure to more accurately capture the full range of responses. In the subsequent psychometric testing phase, those items in the German SScQoL revealed significant deviations from the Rasch model, confirming the problems highlighted by patients. This suggested the need for revision of the German SScQoL [13]. The need for revision was in the item wording/presentation, response structure and further psychometric testing of the German SScQoL. The aim of this present study was to review the German SScQoL, expand the response structure, and examine content validity, construct validity, unidimensionality, and reliability of the scale.

## Methods

### Design

This study consisted of two phases involving cognitive interviews for clarifying the cultural adaptation and a validation study to establish measurement validity of the adapted tool. In Phase 1, the SScQoL was refined in accordance with the International Society for Pharmacoeconomics and Outcomes Research (ISPOR) guideline [16, 17]. Phase 2, drew on data from the *MANagement Of Systemic Sclerosis (MANOSS)* cross-sectional study carried out in Switzerland [18, 19]. The *MANOSS* project aims to fill existing gaps in SSc care by developing an eHealth-enhanced rare disease chronic care model for SSc patients in Switzerland. Part of the *MANOSS* project involves conducting baseline data of SSc patients before implementing a new model of care (i.e., HRQoL). The *MANOSS* study was reviewed and approved by the responsible Swiss ethics committee in September 2018 (EKNZ 2018‐01206).

### Measures

In phase 1, the original English SScQoL and German translation were compared independently by two researchers from Germany (KH) and Switzerland (AK) respectively. The revised translations of both researchers were discussed until consensus was achieved. Subsequently, an expert committee (MN, DN, KH, AK) expanded the response structure for items 1, 3–5, 7–14, 16–17, 19–22, and 25–29 from dichotomous (true/not true) into polytomous (‘always’, ‘usually’, ‘sometimes’, ‘never’) responses. The final version was back-translated into English language by a professional translator. In cognitive interviews, a convenience sample of patients with SSc completed the new version while ‘*thinking aloud*’ and commented on relevance of the items and the response structure. Briefly, participants were encouraged to read all SScQoL items while verbalizing their thoughts concurrently. Additionally, cognitive interviews were used for cognitive debriefing to identify problems interpreting items and response options in the intended way [20, 21]. This approach has shown to be appropriate for quality of life items and for detecting unanticipated problems in participant response behaviour with minimal interviewer-imposed bias [20, 22].

In phase 2, the validation study, German-speaking SSc patients of the *MANOSS* cross-sectional survey (March–August 2019) completed the revised (polytomous) SScQoL [18]. Participants completed either a paper format version and returned it by mail or completed the revised SScQoL in a web-based format. Participants provided sociodemographic data (sex, age, education, employment status), self-reported disease information (subset: lSSc, dSSc, Overlap syndrome^1^ or unknown), and disease duration.

### Participants

For phase 1, a convenience sample of six SSc patients spanning a range of SSc disease severity/experiences and with varied educational levels was recruited from a Swiss University hospital (Inselspital, Bern, Switzerland), a German University hospital (Medizinische Hochschule Hannover, Germany) and a German outpatient rheumatology clinic (rheumapraxis an der hase, Osnabrück, Germany). They were included if they (1) had an SSc diagnosis assured by a physician, were (2) adult (> 18 years), and (3) understood the German language. They were asked to assess the face validity of the revised SScQoL. For phase 2, patients were recruited according to the *MANOSS* protocol [18]. Patients were recruited from four Swiss University hospitals, one regional (cantonal) hospital, rheumatology outpatient clinics, and the Swiss SSc patient association. Participants were included if they were (1) adult (> 18 years), (2) received care in the Swiss healthcare system, and (3) understood the German language.

### Data analysis

Cognitive interview data were analysed by an expert committee (AK, MN, KH, AR, DN) who made final decisions on the revised German SScQoL. For phase 2, the Swiss sample is described using descriptive statistics including frequencies, percentages, median, interquartile range (IQR), mean and standard deviation (SD). To assess whether the German SScQoL had retained its validity and reliability following the revision process, we used Rasch analysis—a psychometric testing technique that compares collected data with the Rasch model [19, 23]. Originally used in education, Rasch analysis has gained wide acceptance in the health sciences [19]. Fit to the Rasch model implies construct validity, reliability and statistical sufficiency of the item scores [23]. Rasch analysis was performed using RUMM2030 software (Perth, WA: RUMM Laboratory Pty Ltd) with the Master’s Partial Credit Model (PCM), a polytomous generalization of the Rasch model, which does not impose a common threshold structure across all items [19].

First, each of the 29 SScQoL items was assessed for ‘fit’ to the Rasch model to examine how the 29-item tool works as a scale. Second, items were grouped into the 5-domains established in the previous cross-cultural validation study (Ndosi et al.) and tested as a 5-subscale measure of quality of life in SSc. Detailed descriptions of the Rasch model requirements are published elsewhere [19]. Briefly, model fit was tested by Chi-square-based fit statistics comparing differences between observed values and those expected by the model, i.e., (i) item-person interaction statistics, expressed as a Z score are expected to have a mean of zero (range − 2.5 to 2.5) and standard deviation (SD) of one and (ii) a non-significant Chi-square probability. In addition to fit statistics, internal consistency (inter-relatedness of items) demonstrating scale reliability was assessed using Person Separation Index (PSI) which functions in the same way as Cronbach’s alpha but is expressed in a logit scale. A minimal PSI value of 0.7 is accepted for assessment at a group level and 0.85 for individual level [19]. Another type of reliability, the invariance of the tool (also known as differential item functioning—DIF) was established by testing if there was a response bias by different subgroups of patients based on personal and clinical characteristics (sex, age, educational background and type of SSc). DIF is tested by assessing item-trait Chi-square interaction statistic and a non-significant Bonferroni-adjusted probability to determine if the tool performs consistently across different subgroups of patients. Principal component analysis and* t* test-based method was used to assess (strict) unidimensionality of the scale as previously described [24]. This test compares two sets of items hypothesized to represent low levels and high levels of the construct (quality of life), selected based on the correlation between items and the first residual factor. The difference in estimates for each person are compared using an independent t-test. Unidimensionality is confirmed if  ≤ 5% of t tests are significant or if the lower bound of a binomial 95% CI of the observed proportion overlap 5% [24]. A *p* value of < 0.05 was considered significant—except when a Bonferroni adjustment was applied to account for multiple testing (i.e. 0.05/number of tests). IBM® SPSS® Version 26. Armonk, NY: IBM Corp. and RUMM2030 software, Perth, WA: RUMM Laboratory Pty Ltd were used for all quantitative analyses.

## Results

### Cognitive interviews

A convenience sample of German-speaking patients with SSc from Germany (n = 4) and Switzerland (n = 2) completed the new SScQoL version using “thinking aloud” techniques for cognitive interviews (Additional File 1). Patients identified some problems with item wording and the remaining dichotomous (true/not true) responses. Specifically, participants desired greater differentiation beyond a binary choice (i.e. addition of ‘sometimes’). Based on patient feedback, the expert committee (AK, MN, KH, AR, DN) decided to expand the 4-point response structure to all items. A summary of issues raised for each item during back-translation and cognitive interviews is presented in Additional File 1.

### Cross-sectional validation study

#### Patient characteristics

The validation study sample comprised 78 Swiss-German patients with SSc. They had a median self-reported disease duration (i.e. date of diagnosis) of 8 years (IQR 4–13 years) and the majority, 58/78 (74.7%) were women. Participants’ sociodemographic data are summarized in Table 1. The descriptive results including frequency and distribution of all items are shown in Additional File 2.

**Table 1:** Validation study: Participant characteristics (n = 78)

Characteristic	n (%)
**Instrument format**	
Online survey	25 (32.1%)
Paper survey	53 (67.9%)
**Sex**	
Female	59 (75.6%)
Male	17 (21.8%)
Not reported	2 (2.6%)
**Age** [years, median (IQR)]	61 (49–71)
**Disease duration, self-reported** [years, median (IQR)]	8 (4–13)
Not reported	5
**Disease subset, self-reported**	
Limited cutaneous systemic sclerosis (lSSc)	28 (35.9%)
Diffuse cutaneous systemic sclerosis (dSSc)	22 (28.2%)
Overlap syndrome^1^	3 (3.9%)
Don’t’ know	20 (25.6%)
Not reported	5 (6.4%)
**Comorbidities, self-reported**	
Gastrointestinal problems	46 (58.2%)
Osteoarthritis	32 (40.5%)
Backpain	31 (39.2%)
Lung problems	28 (35.4%)
High blood pressure	24 (30.4%)
Heart problems	22 (27.8%)
Depression	12 (15.2%)
Anemia or other blood problems	10 (12.7%)
Liver problems	9 (11.4%)
Diabetes	5 (6.3%)
Kidney problems	3 (3.8%)
**Marital status**	
Single	11 (14.1%)
Married/cohabiting	52 (66.7%)
Divorced, separated, or widowed	13 (16.7%)
Not reported	2 (2.5%)
**Education**	
Tertiary level (e.g. university of applied science)	32 (41.1%)
Upper secondary (e.g. Baccalaureate schools)	34 (43.5%)
Compulsory (e.g. high school)	10 (12.8%)
No completed school education or vocational training	1 (1.3%)
Not reported	1 (1.3%)
**Employment**^2^	
Employed	38 (48.7%)
Working full time (80–100% employed)	17/38 (21.8%)
Working part time (less than 80% employed)	21/38 (26.9%)
Looking for work	4 (5.1%)
In training (student, vocational education)	7 (9.0%)
Retired	19 (24.4%)
On disability or sick leave	10 (12.8%)
Not reported	1 (1.3%)

^1^*Overlap syndrome:* Condition in which patients have concurrent clinical manifestations of multiple distinct immune diseases (e.g. overlap between systemic sclerosis and rheumatoid arthritis) ^2^*Multiple answers were allowed*

#### Response scale structure

After expanding the response structure, item characteristic curves (ICC) revealed that 22/29 displayed ordered thresholds suggesting that the response categories represented by the thresholds were ordered from low to high (quality of life) as expected (Additional File 3). Collapsing some categories and rescoring items with disordered thresholds improved the individual item fit but not the overall scale.

#### Fit to the model

Item fit statistics for individual items are shown in Table 2a. Most individual items, appeared to adequately fit the model limits (residuals within the − 2.5 to 2.5 range) with non-significant Chi-Square Bonferroni-adjusted probability (*p* = 0.0017). The sole exception was item 29 with a fit residual of − 2.573. This may have impacted on the overall validity of the scale (summary statistics indicating deviation from the model) as shown in Table 3 (Chi-Square = 52.198, *DF* = 29, *p* = 0.005). When the items were grouped in their respective domains and analysed (Table 2b), each domain was found to adequately fit the model. Summary statics indicate the 5-domain structure has adequate fit to the model (Chi-Square = 5.269, *df* = 5, *p* = 0.384) (Table 3). The reliability of the scale was high (PSI = 0.915). The proportion of significant t-tests was < 5% (i.e. 0.0649, 95% CI 0.016–0.114) supporting the unidimensionality of the scale.

**Table 2:** Fit statistics for individual items and subscales

Item	Location	SE	Fit residual	*DF*	Chi-Square	*DF*	*p* value*
a: **Individual item fit statistic**							
Item 1	− 0.0690	0.1980	0.7720	71.30	0.7350	1	0.3913
Item 2	0.8040	0.2000	− 0.1080	71.30	0.0950	1	0.7575
Item 3	− 0.5990	0.2040	0.7390	69.45	0.4630	1	0.4961
Item 4	− 0.5850	0.2080	1.4080	70.37	1.7500	1	0.1859
Item 5	0.5520	0.1870	2.0380	71.30	6.9000	1	0.0086
Item 6	− 0.7860	0.2240	0.4820	69.45	0.0430	1	0.8361
Item 7	0.5300	0.1650	− 0.0030	70.37	0.0590	1	0.8088
Item 8	0.3000	0.1700	− 1.5930	70.37	0.7270	1	0.3940
Item 9	0.1440	0.1760	1.4490	70.37	0.5550	1	0.4562
Item 10	0.1980	0.1720	− 1.0090	70.37	0.8430	1	0.3586
Item 11	− 0.0240	0.1880	− 0.0300	71.30	1.1780	1	0.2777
Item 12	1.9560	0.1660	2.0370	71.30	1.6340	1	0.2011
Item 13	0.0080	0.1910	− 1.3440	69.45	1.2680	1	0.2602
Item 14	1.3880	0.1640	− 0.1000	70.37	0.0840	1	0.7720
Item 15	0.5680	0.1770	− 0.5930	69.45	1.2140	1	0.2705
Item 16	− 0.1550	0.1860	− 0.1190	68.52	0.0040	1	0.9525
Item 17	− 0.1520	0.1970	− 0.1290	69.45	0.5860	1	0.4438
Item 18	1.2400	0.1670	0.5370	70.37	0.0130	1	0.9100
Item 19	− 1.6870	0.2340	− 1.2330	70.37	4.8620	1	0.0274
Item 20	− 0.0900	0.1790	0.6500	70.37	1.6790	1	0.1950
Item 21	− 2.0530	0.2340	− 1.4820	69.45	7.6950	1	0.0055
Item 22	0.8940	0.1670	− 0.1830	70.37	0.0140	1	0.9060
Item 23	1.0750	0.1620	1.6730	70.37	5.2190	1	0.0223
Item 24	− 1.8890	0.2630	− 0.2340	69.45	0.0220	1	0.8822
Item 25	− 0.6260	0.1870	0.4180	70.37	0.2140	1	0.6437
Item 26	0.3040	0.1910	− 1.2790	70.37	2.9170	1	0.0876
Item 27	− 0.6810	0.2030	− 1.3730	71.30	4.9500	1	0.0261
Item 28	− 0.5330	0.2000	− 1.2820	70.37	1.2640	1	0.2609
Item 29	− 0.0350	0.1690	− **2.5730**	71.30	4.2360	1	0.0396
b: **Fit statistics for each domain (subscale)**							
Function	0.618	0.129	0.215	51.65	1.339	1	0.2473
Emotional	− 0.075	0.079	− 1.05	49.4	0.202	1	0.6528
Sleep	− 0.294	0.172	0.236	53.15	0.038	1	0.8445
Social	− 0.16	0.109	− 0.22	50.15	1.933	1	0.1644
Pain	− 0.089	0.19	− 0.13	51.65	1.757	1	0.1850

DF, degree of freedom; SE, standard error; *p* value*, Bonferroni adjusted p-value = 0.05/number of tests (items), Numbers in bold suggest deviation from the model

**Table 3:** Summary fit statistics

Analysis	Item Fit Residual		Person Fit Residual		Chi Square Interaction				Unidimensionality
	Mean	SD	Mean	SD	Value (*df*)	*p*	PSI	*N*	Independent t-tests (95% CI)
Individual items	− 0.0849	1.1739	− 0.19	1.313	51.2238	0.0066	0.990	77	0.256 (0.208 to 0.305)
Five domains	0.1019	0.9283	− 0.25	0.917	5.3426	**0.3755**	0.915	77	0.0649 (**0.016** to 0.114)
*Model fit*	*0*	*1*	*0*	*1*		** > *0.05***	> *0.7*		*Lower bound 95%CI* < *0.05*

SD, Standard deviation; df, degrees of freedom; PSI, Person separation index

#### Targeting of persons and items

**Fig. 1:**
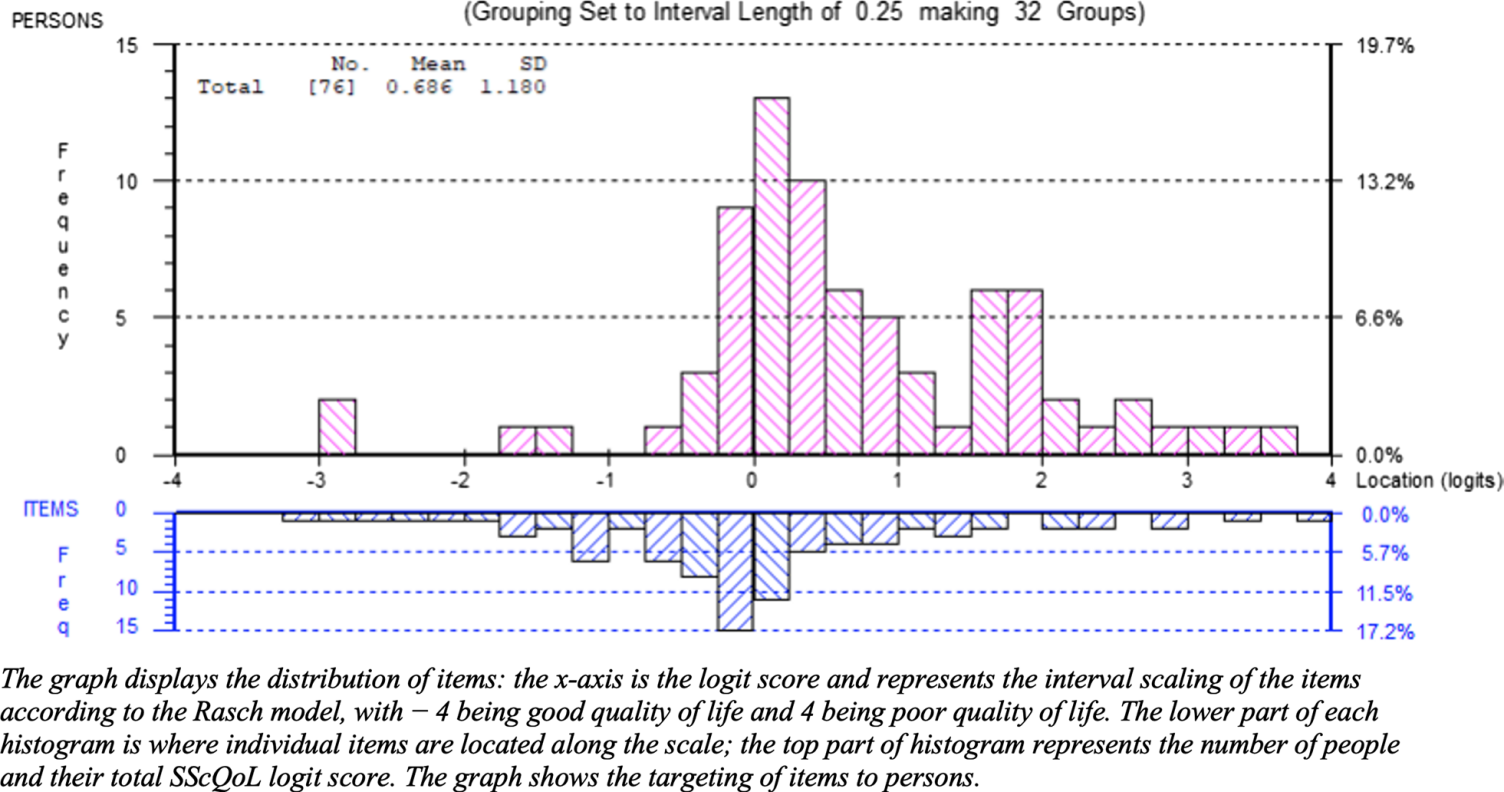
Person-item distribution for all 29 items of the German Systemic Sclerosis Quality of Life Questionnaire (SScQoL)

The revised 29-item German SScQoL version integrating a 4-point response option for all items was shown to cover the full range of participants’ quality of life. The person–item threshold distribution (Fig. 1) depicts that the items are well mapped against all persons.

#### Invariance of the SScQoL

The test of invariance found that there were no significant DIF by any personal characteristics (age, sex, education level) or disease subcategory and disease duration. The results of DIF analysis are presented in Additional Files 4 and 5.

#### Testing the fit of the dichotomized scale

As the response structure of the scale has been expanded to 4 responses, comparison of measures with other countries would require a cross-cultural measurement equivalence which may first require dichotomizing responses of the revised scale. For all items, collapsing categories 1, 2 and 3 vs category 4 provided the best model fit in individual items (domains) and the summary statistics (Additional File 6).

## Discussion

In the present study, we revised the German SScQoL with the aim to linguistically review the German SScQoL, expand the response structure, and used Rasch analysis to examine construct validity, unidimensionality, and reliability. Overall, the scale was well targeted, had high internal consistency, and worked consistently across patients with varied demographic and clinical characteristics. The present data suggest the revised German SScQoL can now be used with confidence in German-speaking countries.

Cognitive interviews included patients from Germany and Switzerland to gain an understanding of how well patients comprehend the concepts intended by the items and how the new response structure worked for them. Cognitive interviews and subsequent expert discussions revealed translation and language issues that are essential for using the SScQoL in all four German-speaking countries (Austria, Germany, Liechtenstein, Switzerland). We made minor linguistic changes enabling use across German-speaking countries. The initial validation study [13] identified ten items that patients found too restrictive and also lacked fit to the Rasch model. In the present study, cognitive interviews informed modification of the response structure thereby facilitating more accurate responses. Polytomous responses (‘always’, ‘usually’, ‘sometimes’, ‘never’) were applied to all items—although linguistically, this may not always make sense (e.g. for item Q23: *‘I have had to stop some of my hobbies’*). Importantly, there is no definitive consensus on the most appropriate translation or questionnaire response format for measuring HRQoL [15]. In the present study, expanding all items to a uniform, 4-point response structure improved the validity and reliability of the German SScQoL. Although there is not necessarily semantic or linguistic equivalence with the English SScQoL, expert meetings and cognitive interviews support conceptual equivalence between the English and German versions.

Rasch analysis confirmed that measurement properties (construct validity, reliability, and unidimensionality) of the SScQoL were retained following its revision in German. Similar to the prior multinational cross-cultural validation using Rasch analysis [13], the SScQoL demonstrated adequate fit when the items were grouped into the five domains. Validity, reliability and unidimensionality of the German SScQoL was demonstrated. Additionally, the tool had good targeting for patients with different levels of HRQoL and was shown to be free of response bias for age, sex, education level, disease subcategory, and disease duration (DIF analysis shown in Additional Files 4 and 5). Overall, fit to the Rasch model confirmed that the measurement properties of the revised German SScQoL version integrating a 4-point response option were retained.

Having a 4-point response structure means that the total score will range from 0 to 87 (i.e. scoring always = 3, usually = 2, sometimes = 1, never = 0) which differs from the original SScQoL (score range: 0–29). For interoperability in research settings, the polytomous scale could be re-scored dichotomously (i.e. ‘always’, ‘usually’ or ‘sometimes’ = ‘true’/1, ‘never’ = ‘not true’/0). We tested this scoring approach and it showed adequate fit to the model (Additional File 6). Instructions for scoring are included in Additional File 7.

The study has several limitations. First, the validation was only planned when the *MANOSS* project was already established and did not allow for confirmation of the self-reported diagnosis, multiple measurement points and multinational validation [18]. For the cognitive interviews, only six Swiss and German patients were included. Including more patients (i.e. from Austria and Liechtenstein) would have been ideal, although this was not possible. Field testing with more patients from all German-speaking countries could further improve the linguistic presentation of the SScQoL, although we believe conceptual equivalence is more important [15]. Our validation sample only included Swiss German-speaking patients. Thus, caution is warranted when attempting to extend findings to other German-speaking populations. Further studies should include patients from Austria, Germany and Liechtenstein to confirm the robustness of the German SScQoL and ensure transferability. Last, while the instrument is well targeted and the sample size adequate for its validation [25], calibration of the scale into interval-level (transformed) scores was beyond the scope of this study. Future work should include establishing responsiveness of the SScQoL and calibration or cross-cultural comparability studies using data from other European countries.

## Conclusions

The data presented herein contributes to the existing literature through the successful revision and validation of the SScQoL, with a new 4-point response structure for the German speaking context. These data are relevant to the broader rare disease research community as they demonstrate that cognitive interviews and Rasch analysis can improve the psychometric properties of PROMs while enabling interoperability of findings. Further cross-cultural validity tests are required to fully demonstrate measurement equivalence with other SScQoL versions, thereby enabling broad, multilinguistic comparison and data pooling. Beyond research, the new German SScQoL is a valid measure that can be used with confidence in clinical practice. The new version of the SScQoL can be obtained at https://doi.org/10.5518/325.

## Supplementary Information

**Additional File 1**. Back-translation, issues and agreements for each item of the German Systemic Sclerosis Quality of Life Questionnaire (SScQoL).

**Additional File 2**. Frequency and level of quality of life of all items of the new German Systemic Sclerosis Quality of Life Questionnaire (SScQoL) version (N = 78).

**Additional File 3**. Item characteristic curves (ICC) for all items.

**Additional File 4**. Differential item functioning (DIF) analysis A.

**Additional File 5**. Differential item functioning (DIF) analysis B.

**Additional File 6**. Testing the dichotomized responses.

**Additional File 7**. Scoring instructions for the new German version of the Systemic Sclerosis Quality of Life (SScQoL) questionnaire.

## Data Availability

The datasets generated and/or analysed during this study are included in this published article and its supplementary files, or can be made available from the corresponding author on reasonable request.
