# Supplementary material for: A rare disease patient-reported outcome measure: revision and validation of the German version of the Systemic Sclerosis Quality of Life Questionnaire (SScQoL) using the Rasch model
Source: Orphanet J Rare Dis. 2021 Aug 9;16:356. doi: 10.1186/s13023-021-01944-9 (PMC8351336; doi:10.1186/s13023-021-01944-9)
Supplement: Supplementary file 1 — Additional File 1. Back-translation, issues and agreements for each item of the German Systemic Sclerosis Quality of Life Questionnaire (SScQoL). [file 13023_2021_1944_MOESM1_ESM.pdf]

Additional file 1 Back-translation, issues and agreements for each item of the German Systemic Sclerosis Quality of Life Questionnaire (SScQoL)

Item	English Original	German Original	Back-translation 1 (BT1)	German revised	Answer options	Back-translation 2 (BT2)	Issues raised in cognitive interviews	Agreement
+1	I can’t do anything without really thinking it through	Ich kann nichts tun, ohne vorher gründlich drüber nachzudenken	I can't do anything without thinking about it first	No changes	Immer, meistens, manchmal, nie	I can't do anything without thinking it through in advance		
2	It’s always on my mind	Die Erkrankung ist ständig in meinen Gedanken	The disease is constantly in my mind	No changes	No changes	My illness is always on my mind	More response options are requested.	Add response options
°+3	I worry that I let people down	Ich befürchte/ sorge mich, dass ich andere Menschen enttäusche	I fear/ worry that I will disappoint other people	Ich mache mir Sorgen, dass ich andere Menschen im Stich lasse	Immer, meistens, manchmal, nie	I worry about letting other people down		
*+4	My condition makes me angry	Mein derzeitiger Gesundheitszustand macht mich wütend	My current state of health makes me angry	No changes	Immer, meistens, manchmal, nie	My current state of health makes me very angry	Questions 4 and 5 are very similar.	No changes
°+5	I get upset when I can’t do things	Ich rege mich auf, wenn ich etwas nicht mehr selbst tun/ erledigen kann	I get upset when I can't do something by myself anymore	Ich rege mich auf, wenn ich etwas nicht mehr tun kann	Immer, meistens, manchmal, nie	I get upset when there is something I can't do anymore	Questions 4 and 5 are very similar.	Grammatical changes
6	I often get frustrated	Ich bin oft frustriert	I am often frustrated	No changes	No changes	I often get frustrated	More response options are requested.	Add response options

+7	I cannot rely on how I will be tomorrow	Ich kann mich nicht darauf verlassen, wie es mir am nächsten Tag gehen wird	I can't count on how I'm gonna feel the next day	No changes	Immer, meistens, manchmal, nie	I can't tell how I'll be feeling tomorrow		
°+8	I feel like I'm fighting all the time	Ich fühle mich wie in einem ständigen Kampf	I feel like I'm in a constant battle	Ich fühle mich, als ob ich ständig kämpfen würde	Immer, meistens, manchmal, nie	I feel like I'm fighting a constant battle	The question is too theatrical and hard to answer.	No changes possible
*+9	My condition means I have disturbed sleep	Durch meine Erkrankung habe ich Schlafstörungen	My illness has caused me to have trouble sleeping	No changes	Immer, meistens, manchmal, nie	Because of my illness I have trouble sleeping	Question 9 and 20 are similar.	No changes possible
°+10	It has affected me a lot socially	Die Krankheit beeinträchtigt mein Sozialleben sehr	The disease severely restricts my social life	Die Erkrankung beeinträchtigt mein Sozialleben sehr	Immer, meistens, manchmal, nie	My illness severely restricts my social life		"It" is always changed into „Erkrankung"
°*+11	It has affected the health of people around me	Sie hat Einfluss auf das Befinden der Menschen in meinem Umfeld	It has an influence on the well-being of the people around me	Die Erkrankung hat Einfluss auf das Befinden der Menschen in meinem Umfeld	Immer, meistens, manchmal, nie	My illness influences the sense of well-being of the people around me	What does "It" mean?<br>Question 11 and 13 are very similar.<br>Hard to answer.	"It" is always changed into „Erkrankung"
*+12	My hands don't work as well as they did	Meine Hände funktionieren nicht mehr so gut wie früher	My hands don't work as well as they used to	No changes	Immer, meistens, manchmal, nie	I can't use my hands as well as I used to	Hard to answer.	No changes possible

*+13	It puts a strain on my personal relationships	Die Erkrankung belastet meine persönlichen Beziehungen	The disease strains my personal relationships	No changes	Immer, meistens, manchmal, nie	My illness puts a strain on my personal relationships		
°+14	I need to rest more often	Ich muss mich häufiger ausruhen/ öfter Pausen einlegen	I need to rest more often/ take breaks more often	Ich muss mich häufiger ausruhen	Immer, meistens, manchmal, nie	I have to take more frequent breaks		
*15	Any sort of activity is difficult	Jede Art von Tätigkeit ist mit Schwierigkeiten verbunden	Every type of activity is associated with difficulties	No changes	No changes	Every activity involves some kind of difficulty	It feels wrong to answer with correct or wrong.<br>The question is unclear.	No changes possible<br>Add response options
°+16	I avoid certain social situations because I am embarrassed	Ich vermeide manchmal gesellschaftliche Situationen, um mich nicht in Verlegenheit zu bringen	I sometimes avoid social situations so as not to embarrass myself	Ich vermeide gewisse gesellschaftliche Situationen, um mich nicht in Verlegenheit zu bringen	Immer, meistens, manchmal, nie	I avoid getting into certain situations with others to prevent embarrassment		
+17	I take to heart things which wouldn't have worried me before	Ich nehme mir Dinge zu Herzen, die mich früher nicht bedrückt hätten	I take things to heart that wouldn't have bothered me before	No changes	Immer, meistens, manchmal, nie	There are things that didn't used to bother me but now really get me down		
18	Life is just not what it was	Das Leben ist einfach nicht mehr wie früher	Life just isn't the same anymore	No changes	No changes	Life just isn't like it used to be	One patient is annoyed by the many similar questions.	No changes possible<br>Add response options

°*+19	I can’t cope at all	Ich komme mit der Erkrankung irgendwie nicht zurecht	I don't know how I'm coping with the disease	Ich komme überhaupt nicht zurecht	Immer, meistens, manchmal, nie	I’m simply not able to cope	The question is "dumbly asked", what does this refer to? What can you not cope with?	No changes possible
*+20	Sleeping badly has affected me a lot	Schlecht zu schlafen beeinträchtigt mich sehr	Sleeping badly affects me very much	No changes	Immer, meistens, manchmal, nie	Not sleeping well is a major problem for me	Question 9 and 20 are similar.	No changes possible
+21	I feel very isolated	Ich fühle mich sehr isoliert	I feel very isolated	No changes	Immer, meistens, manchmal, nie	I feel very isolated		
°+22	Household tasks can be a problem	Hausarbeiten können ein Problem darstellen	Housework can be a problem	Hausarbeiten können ein Problem sein	Immer, meistens, manchmal, nie	Household tasks can pose problems		
23	I have had to stop some of my hobbies	Ich musste einige meiner Hobbies aufgeben	I had to give up some of my hobbies	No changes	No changes	I have had to give up some of my hobbies		Add response options
°24	I feel guilty at being ill	Ich habe Schuldgefühle wegen der Krankheit	I feel guilty about the illness	Ich fühle mich schuldig, krank zu sein	No changes	I feel guilty for being ill	Question is stupid and needless.	No changes possible
							More response options are requested.	Add response options

°*+25	I struggle to wash myself as I would like	Ich habe Schwierigkeiten mich selbst so zu waschen, wie ich gerne würde	I have trouble washing myself the way I'd like	Ich habe Schwierigkeiten mich selbst so zu waschen, wie ich gerne möchte	Immer, meistens, manchmal, nie	I try to wash myself as I was used to doing before	Question is difficult to understand.	No changes possible
+26	Pain limits what I can do	Schmerzen schränken mich in meinem Handeln ein	Pain restricts me in my actions	Die Schmerzen schränken mich in meinem Handeln ein	Immer, meistens, manchmal, nie	Pain restricts my activities		
*+27	I feel helpless	Ich fühle mich hilflos	I feel helpless	No changes	Immer, meistens, manchmal, nie	I feel helpless		
+28	Pain tires me out	Schmerzen erschöpfen/ ermüden mich	Pain exhausts/ tires me out	Die Schmerzen laugen mich aus	Immer, meistens, manchmal, nie	The pain wears me out		
+29	I miss being able to sort things out	Ich vermisse es, meine Angelegenheiten selbst erledigen zu können	I miss being able to do my own business	No changes	Immer, meistens, manchmal, nie	I miss taking care of my own affairs		

* Problematic items detected by Ndosi et al. [11]; ° Revised wording; + Revised answer option

Revision and validation of the German version of the Systemic Sclerosis Quality of Life Questionnaire (SScQoL) using Rasch analysis; Quality of Life Research; Kocher, A., Ndosi, N., Denhaerynck, K., Simon, M., Dwyer A.A., Distler, O., Hoeper, K., Künzler-Heule, P., Redmond, A.C., Villiger, P.M., Walker, U.A., Nicca, D.; Institute of Nursing Science (INS), Department Public Health (DPH), Faculty of Medicine, University of Basel, Switzerland, dunja.nicca@unibas.ch
