# Supplementary material for: A rare disease patient-reported outcome measure: revision and validation of the German version of the Systemic Sclerosis Quality of Life Questionnaire (SScQoL) using the Rasch model
Source: Orphanet J Rare Dis. 2021 Aug 9;16:356. doi: 10.1186/s13023-021-01944-9 (PMC8351336; doi:10.1186/s13023-021-01944-9)
Supplement: Supplementary file 2 — Additional File 2. Frequency and level of quality of life of all items of the new German Systemic Sclerosis Quality of Life Questionnaire (SScQoL) version (N = 78). [file 13023_2021_1944_MOESM2_ESM.pdf]

Additional file 2 Frequency and level of quality of life of all items of the new German Systemic Sclerosis Quality of Life Questionnaire (SScQoL) version (N=78)

	**Item name German (D) & English (E)**	**Proportion of answers over scale (n, %)**					**Mean (SD)**	**Median**	**P25**	**P75**	**Min**	**Max**
		**1 Always**	**2 Usually**	**3 Some-times**	**4 Never**	**Not reported**						
**Q1**	D: Ich kann nichts tun, ohne vorher gründlich darüber nachzudenken<br>E: I can't do anything without really thinking it through	5 (6.4%)	7 (9.0%)	40 (51.3%)	26 (33.3%)	0	3.12 (0.82)	3	3	4	1	4
**Q2**	D: Die Erkrankung ist ständig in meinen Gedanken<br>E: It's always on my mind	8 (10.3%)	10 (12.8%)	49 (62.8%)	11 (14.1%)	0	2.81 (0.81)	3	3	3	1	4
**Q3**	D: Ich mache mir Sorgen, dass ich andere Menschen im Stich lasse<br>E: I worry that I let people down	3 (3.9%)	10 (12.8%)	32 (41.0%)	31 (39.7%)	2 (2.6%)	3.20 (0.82)	3	3	4	1	4
**Q4**	D: Mein derzeitiger Gesundheitszustand macht mich wütend<br>E: My condition makes me angry	4 (5.1%)	7 (9.0%)	36 (46.1%)	30 (38.5%)	1 (1.3%)	3.19 (0.81)	3	3	4	1	4
**Q5**	D: Ich rege mich auf, wenn ich etwas nicht mehr tun kann<br>E: I get upset when I can't do things	7 (9.0%)	16 (20.5%)	40 (51.3)	15 (19.2%)	0	2.81 (0.85)	3	2	3	1	4

	Item name German (D) & English (E)	Proportion of answers over scale (n, %)					Mean (SD)	Median	P25	P75	Min	Max
**Q6**	D: Ich bin oft frustriert<br>E: I often get frustrated	3 (3.8%)	5 (6.4%)	38 (48.7%)	30 (38.5%)	2 (2.6%)	3.25 (0.75)	3	3	4	1	4
**Q7**	D: Ich kann mich nicht darauf verlassen, wie es mir am nächsten Tag gehen wird<br>E: I cannot rely on how I will be tomorrow	11 (14.1%)	15 (19.2%)	24 (30.8%)	27 (34.6%)	1 (1.3%)	2.87 (1.06)	3	2	4	1	4
**Q8**	D: Ich fühle mich, als ob ich ständig kämpfen würde<br>E: I feel like I'm fighting all the time	10 (12.8%)	11 (14.1%)	27 (34.6%)	29 (37.2%)	1 (1.3%)	2.97 (1.03)	3	2	4	1	4
**Q9**	D: Durch meine Erkrankung habe ich Schlafstörungen<br>E: My condition means I have disturbed sleep	8 (10.3%)	8 (10.3%)	32 (41.0%)	29 (37.2%)	1 (1.3%)	3.06 (0.95)	3	3	4	1	4
**Q10**	D: Die Erkrankung beeinträchtigt mein Sozialleben sehr<br>E: It has affected me a lot socially	9 (11.5%)	11 (14.1%)	27 (34.6%)	30 (38.5%)	1 (1.3%)	3.01 (1.01)	3	2	4	1	4
**Q11**	D: Die Erkrankung hat Einfluss auf das Befinden der Menschen in meinem Umfeld<br>E: It has affected the health of people around me	6 (7.7%)	11 (14.1%)	33 (42.3%)	28 (35.9%)	0	3.06 (0.90)	3	3	4	1	4

	Item name German (D) & English (E)	Proportion of answers over scale (n, %)					Mean (SD)	Median	P25	P75	Min	Max
**Q12**	D: Meine Hände funktionieren nicht mehr so gut wie früher<br>E: My hands don't work as well as they did	26 (33.3%)	18 (23.1%)	27 (34.6%)	7 (9.0%)	0	2.19 (1.01)	2	1	3	1	4
**Q13**	D: Die Erkrankung belastet meine persönlichen Beziehungen<br>E: It puts a strain on my personal relationships	7 (9.0%)	6 (7.7%)	35 (44.9%)	28 (35.9%)	2 (2.6%)	3.11 (0.90)	3	3	4	1	4
**Q14**	D: Ich muss mich häufiger ausruhen<br>E: I need to rest more often	19 (24.4%)	19 (24.4%)	24 (30.8%)	15 (19.2%)	1 (1.3%)	2.45 (1.07)	3	2	3	1	4
**Q15**	D: Jede Art von Tätigkeit ist mit Schwierigkeiten verbunden<br>E: Any sort of activity is difficult	10 (12.8%)	12 (15.4%)	33 (42.3%)	21 (26.9%)	2 (2.6%)	2.86 (0.98)	3	2	4	1	4
**Q16**	D: Ich vermeide gewisse gesellschaftliche Situationen, um mich nicht in Verlegenheit zu bringen<br>E: I avoid certain social situations because I am embarrassed	5 (6.4%)	10 (12.8%)	28 (35.9%)	32 (41.0%)	3 (3.8%)	3.16 (0.90)	3	3	4	1	4
**Q17**	D: Ich nehme mir Dinge zu Herzen, die mich früher nicht bedrückt hätten<br>E: I take to heart things which wouldn't have worried me before	5 (6.4%)	8 (10.3%)	35 (44.9%)	28 (35.9%)	2 (2.6%)	3.13 (0.85)	3	3	4	1	4

	Item name German (D) & English (E)	Proportion of answers over scale (n, %)					Mean (SD)	Median	P25	P75	Min	Max
**Q18**	D: Das Leben ist einfach nicht mehr wie früher<br>E: Life is just not what it was	17 (21.8%)	16 (20.5%)	30 (38.5%)	14 (17.9%)	1 (1.3%)	2.53 (1.03)	3	2	3	1	4
**Q19**	D: Ich komme überhaupt nicht zurecht<br>E: I can't cope at all	2 (2.6%)	4 (5.1%)	21 (26.9%)	50 (64.1%)	1 (1.3%)	3.55 (0.72)	4	3	4	1	4
**Q20**	D: Schlecht zu schlafen beeinträchtigt mich sehr<br>E: Sleeping badly has affected me a lot	6 (7.7%)	10 (12.8%)	29 (37.2%)	32 (41.0%)	1 (1.3%)	3.13 (0.92)	3	3	4	1	4
**Q21**	D: Ich fühle mich sehr isoliert<br>E: I feel very isolated	1 (1.3%)	6 (7.7%)	21 (26.9%)	48 (61.5%)	2 (2.6%)	3.53 (0.70)	4	3	4	1	4
**Q22**	D: Hausarbeiten können ein Problem sein<br>E: Household tasks can be a problem	13 (16.7%)	19 (24.4%)	26 (33.3%)	19 (24.4%)	1 (1.3%)	2.66 (1.03)	3	2	3	1	4
**Q23**	D: Ich musste einige meiner Hobbies aufgeben<br>E: I have had to stop some of my hobbies	17 (21.8%)	14 (17.9%)	29 (37.2%)	17 (21.8%)	1 (1.3%)	2.60 (1.07)	3	2	3	1	4
**Q24**	D: Ich fühle mich schuldig, krank zu sein<br>E: I feel guilty at being ill	2 (2.6%)	2 (2.6%)	16 (20.5%)	56 (71.8%)	2 (2.6%)	3.66 (0.66)	4.00	3.00	4.00	1.00	4.00
**Q25**	D: Ich habe Schwierigkeiten mich selbst so zu waschen, wie ich gerne möchte<br>E: I struggle to wash myself as I would like	7 (9.0%)	3 (3.8%)	7 (9.0%)	60 (76.9%)	1 (1.3%)	3.56 (0.94)	4.00	4.00	4.00	1.00	4.00

	Item name German (D) & English (E)	Proportion of answers over scale (n, %)					Mean (SD)	Median	P25	P75	Min	Max
**Q26**	D: Die Schmerzen schränken mich in meinem Handeln ein<br>E: Pain limits what I can do	7 (9.0%)	13 (16.7%)	37 (47.4%)	20 (25.6%)	1 (1.3%)	2.91 (0.89)	3	2	4	1	4
**Q27**	D: Ich fühle mich hilflos<br>E: I feel helpless	5 (6.4%)	3 (3.8%)	24 (30.8%)	46 (59.0%)	0	3.42 (0.85)	4	3	4	1	4
**Q28**	D: Die Schmerzen laugen mich aus<br>E: Pain tires me out	5 (6.4%)	6 (7.7%)	28 (35.9%)	38 (48.7%)	1 (1.3%)	3.29 (0.87)	3	3	4	1	4
**Q29**	D: Ich vermisse es, meine Angelegenheiten selbst erledigen zu können<br>E: I miss being able to sort things out	9 (11.5%)	9 (11.5%)	21 (27.0%)	39 (50.0%)	0	3.15 (1.03)	3	3	4	1	4

Kocher, A., Ndosi, N., Denhaerynck, K., Simon, M., Dwyer A.A., Distler, O., Hoeper, K., Künzler-Heule, P., Redmond, A.C., Villiger, P.M., Walker, U.A., Nicca, D.; Institute of Nursing Science (INS), Department Public Health (DPH), Faculty of Medicine, University of Basel, Switzerland, dunja.nicca@unibas.ch
