# Supplementary material for: A rare disease patient-reported outcome measure: revision and validation of the German version of the Systemic Sclerosis Quality of Life Questionnaire (SScQoL) using the Rasch model
Source: Orphanet J Rare Dis. 2021 Aug 9;16:356. doi: 10.1186/s13023-021-01944-9 (PMC8351336; doi:10.1186/s13023-021-01944-9)
Supplement: Supplementary file 3 — Additional File 3. Item characteristic curves (ICC) for all items. [file 13023_2021_1944_MOESM3_ESM.pdf]

Additional file 3 Item characteristic curves (ICC) for all items

Item 1

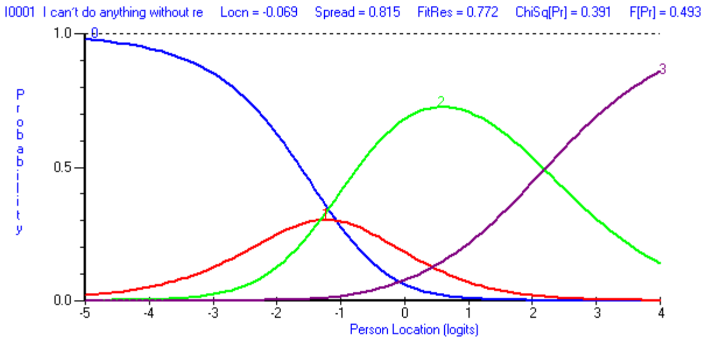

Item 2

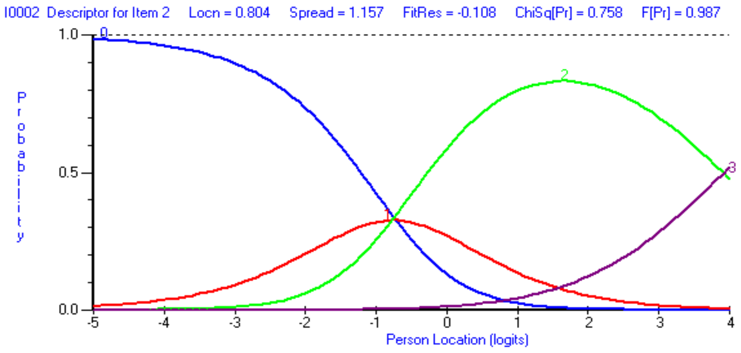

Item 3

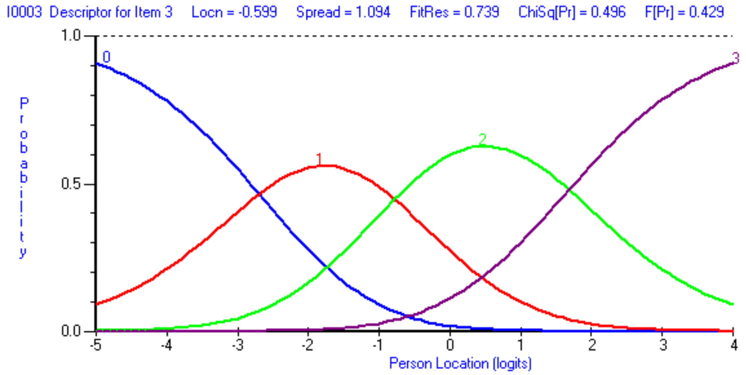

Item 4

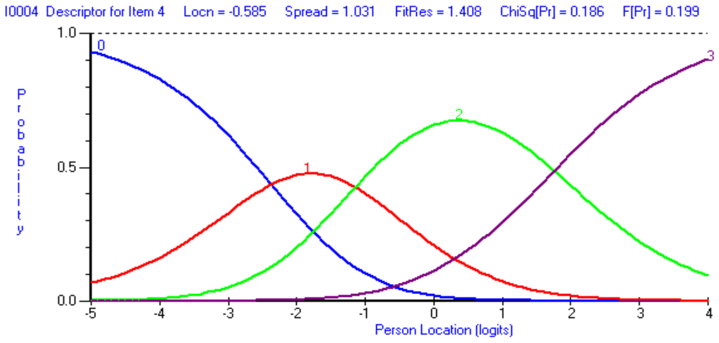

Item 5

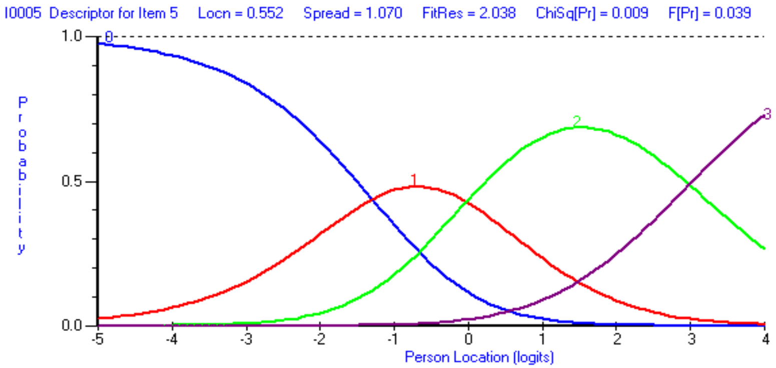

Item 6

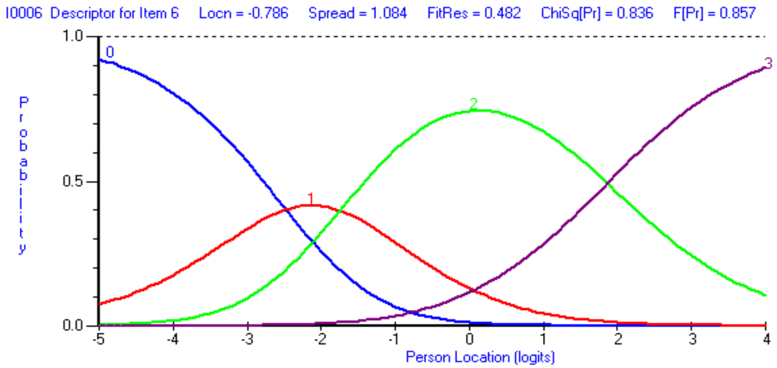

Item 7

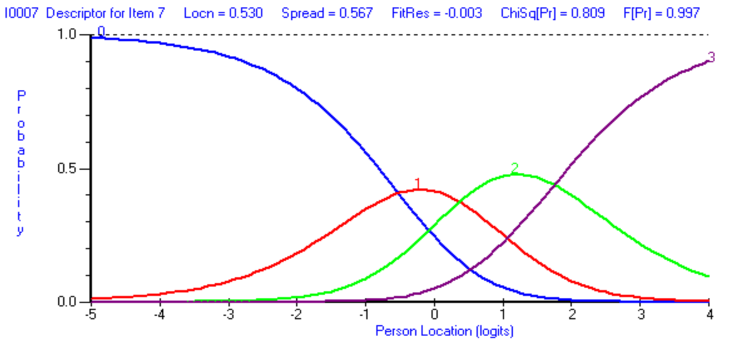

Item 8

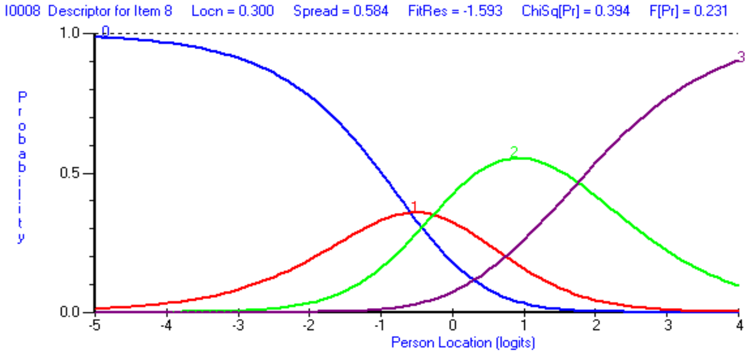

Item 9

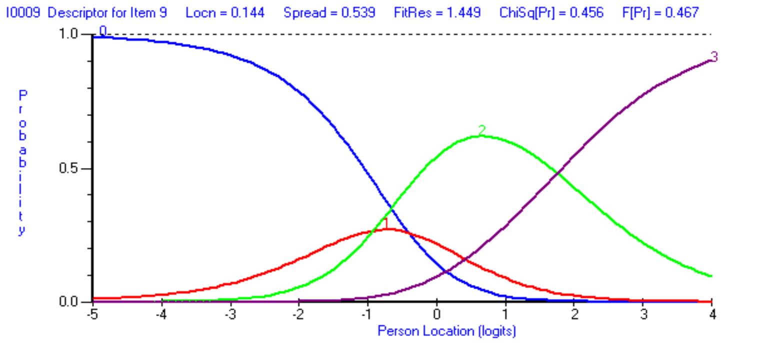

Item 10

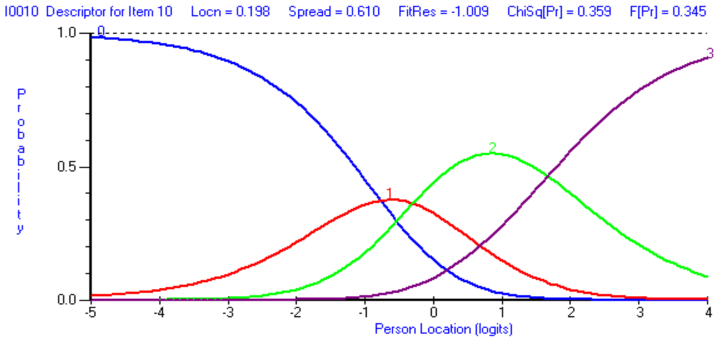

Item 11

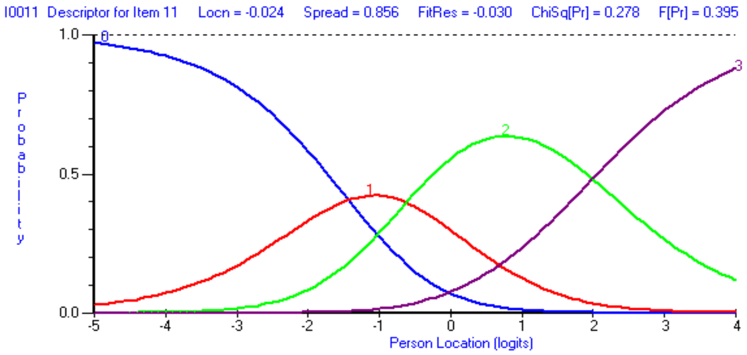

Item 12

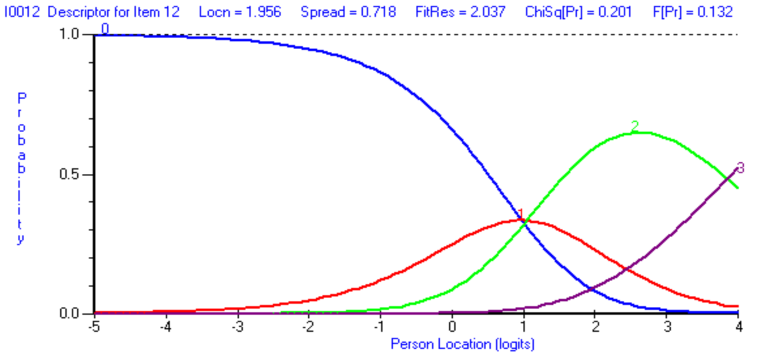

Item 13

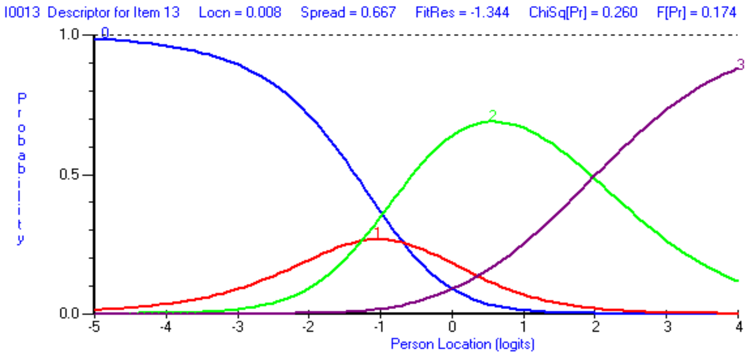

Item 14

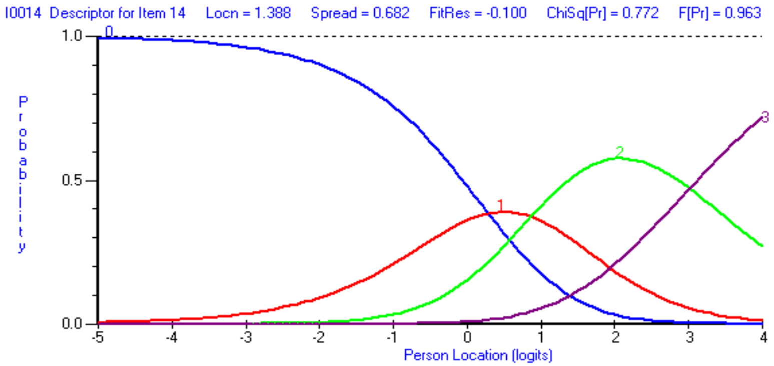

Item 15

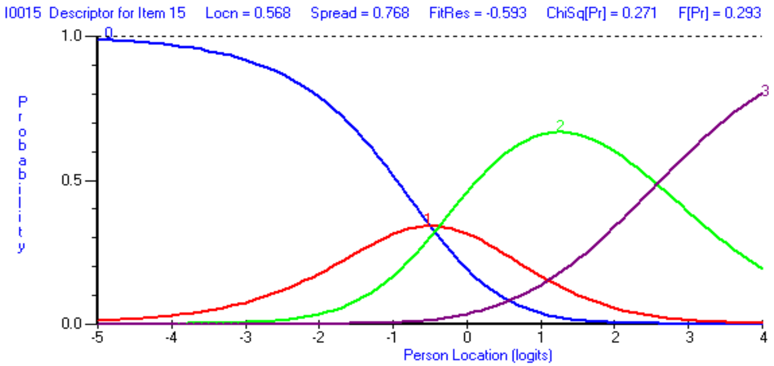

Item 16

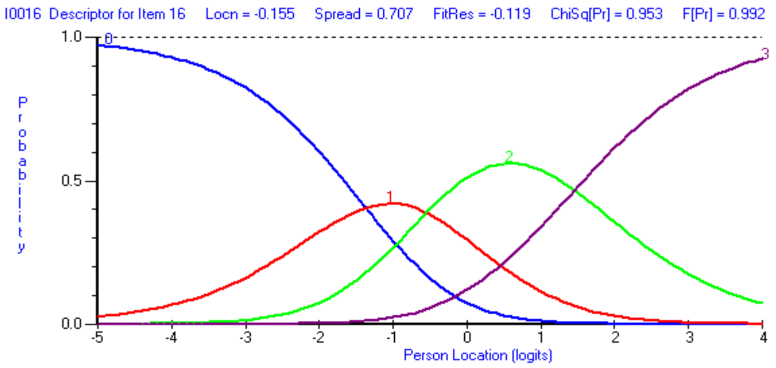

Item 17

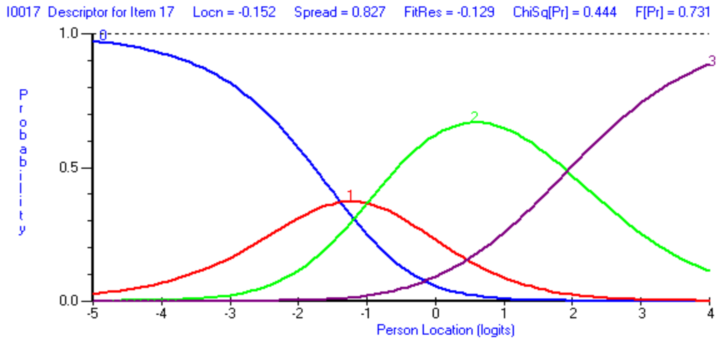

Item 18

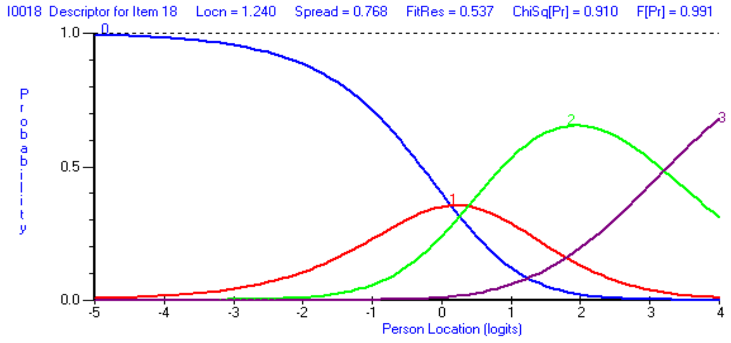

Item 19

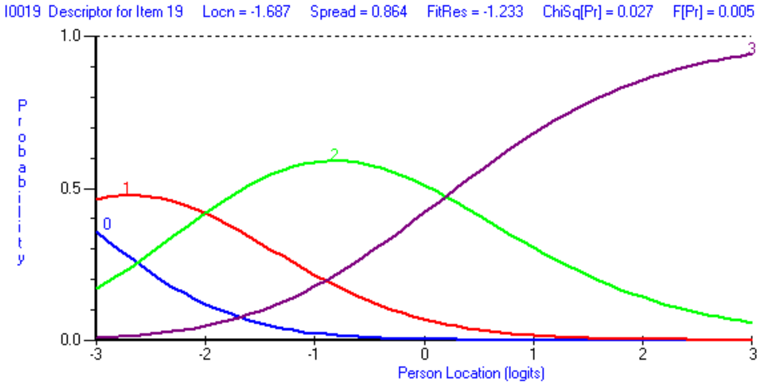

Item 20

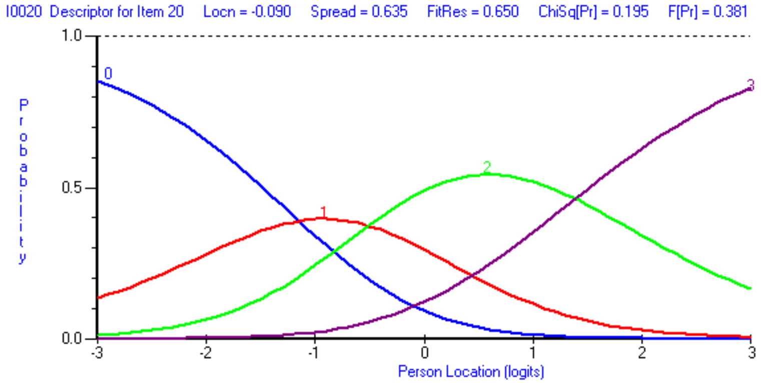

Item 21

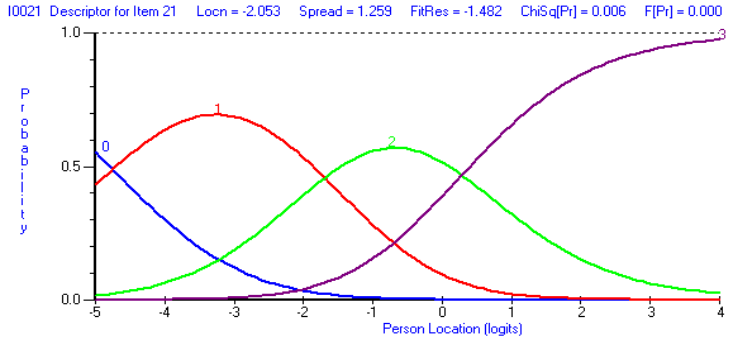

Item 22

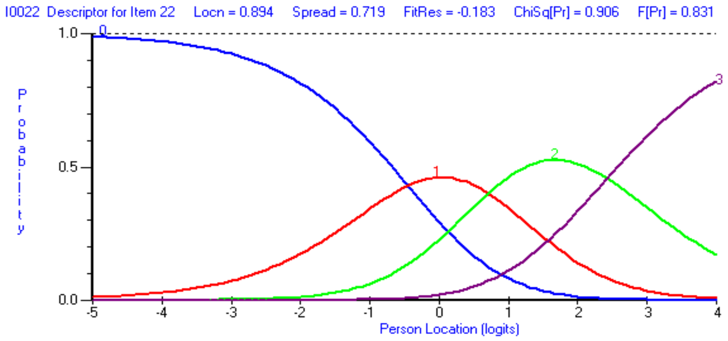

Item 23

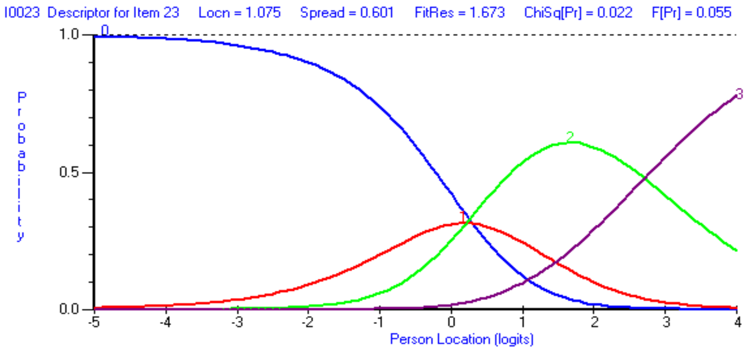

Item 24

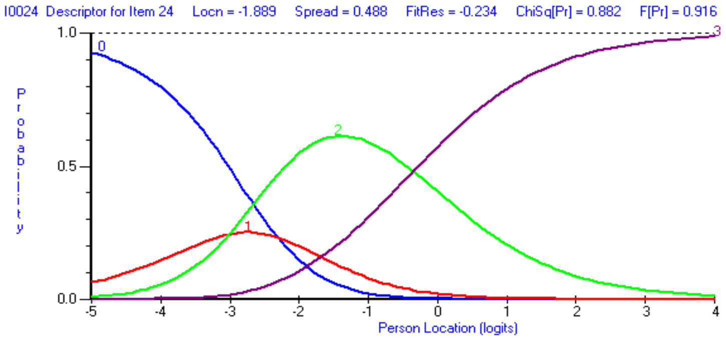

Item 25

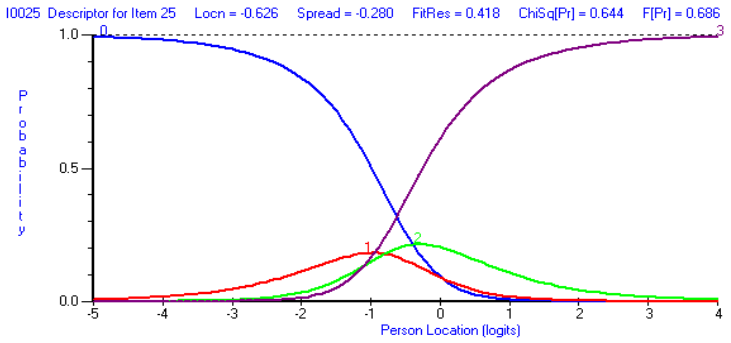

Item 26

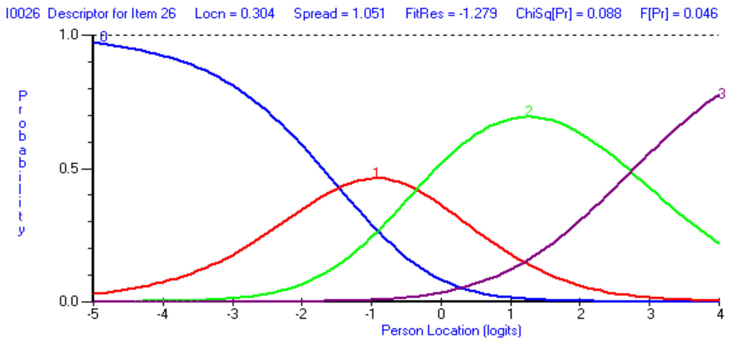

Item 27

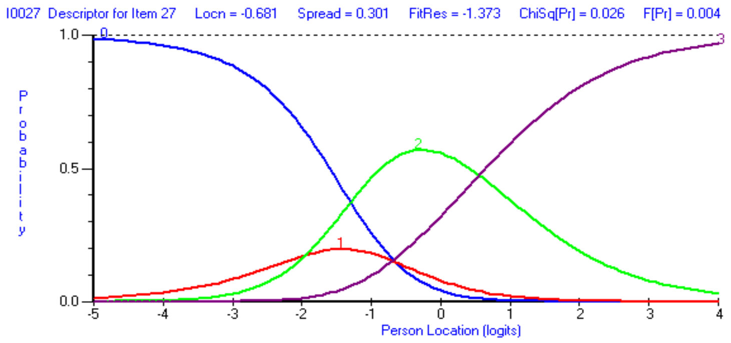

Item 28

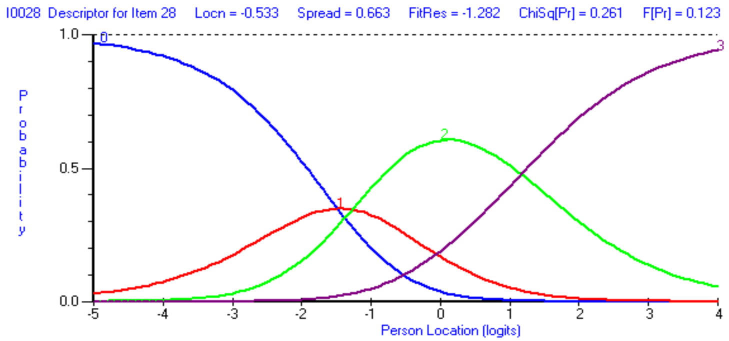

Item 29

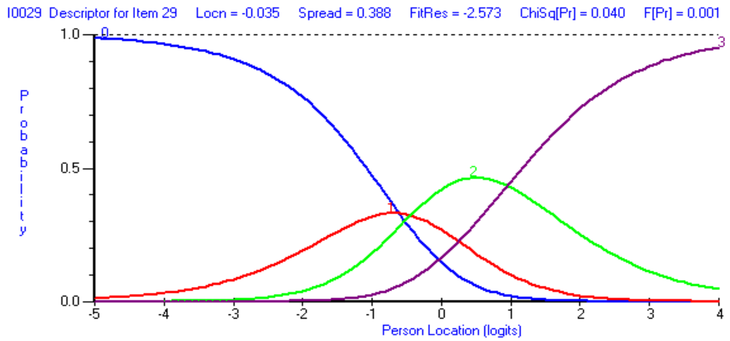

Revision and validation of the German version of the Systemic Sclerosis Quality of Life Questionnaire (SScQoL) using Rasch analysis; Orphanet Journal of Rare Diseases; Kocher, A., Ndosi, N., Denhaerynck, K., Simon, M., Dwyer A.A., Distler, O., Hoeper, K., Künzler-Heule, P., Redmond, A.C., Villiger, P.M., Walker, U.A., Nicca, D.; Institute of Nursing Science (INS), Department Public Health (DPH), Faculty of Medicine, University of Basel, Switzerland, dunja.nicca@unibas.ch
