# Supplementary material for: A rare disease patient-reported outcome measure: revision and validation of the German version of the Systemic Sclerosis Quality of Life Questionnaire (SScQoL) using the Rasch model
Source: Orphanet J Rare Dis. 2021 Aug 9;16:356. doi: 10.1186/s13023-021-01944-9 (PMC8351336; doi:10.1186/s13023-021-01944-9)
Supplement: Supplementary file 4 — Additional File 4. Differential item functioning (DIF) analysis A. [file 13023_2021_1944_MOESM4_ESM.pdf]

Additional file 4 Differential item functioning (DIF) analysis A

	DIF by Sex (M vs F) = Not significant				DIF by Age (62 vs 63+) = Not significant				DIF by education level (7 levels) = Not significant				DIF by disease duration (9 vs 10+) = Not significant				DIF by disese subgroup (lSSc/dSSc/uSSc/Unknown) = Not significant			
Item	MS	F	DF	p-value*	MS	F	DF	p-value*	MS	F	DF	p-value*	MS	F	DF	p-value*	MS	F	DF	p-value*
Item 1	0.1109	0.1001	1	0.7526	1.9376	1.8028	1	0.1837	0.7407	0.6501	6	0.6898	0.0008	0.0006	1	0.9799	0.92143	0.8143	3	0.4907
Item 2	0.9953	1.0416	1	0.3109	2.5012	2.8352	1	0.0967	0.5090	0.4950	6	0.8097	2.8951	3.7588	1	0.0566	1.33392	1.4829	3	0.2276
Item 3	0.3045	0.3015	1	0.5847	2.0173	1.9211	1	0.1703	3.1871	3.3784	6	0.0061	0.3037	0.2742	1	0.6023	0.08655	0.0746	3	0.9734
Item 4	0.0533	0.0414	1	0.8394	5.6203	4.6388	1	0.0348	1.9128	1.6386	6	0.1516	0.5296	0.4328	1	0.5128	1.30932	1.0011	3	0.3983
Item 5	0.9894	0.7703	1	0.3831	0.1466	0.1071	1	0.7445	0.5295	0.3586	6	0.9022	0.0002	0.0002	1	0.9889	1.61076	1.4188	3	0.2454
Item 6	0.1029	0.0946	1	0.7593	2.6132	2.5456	1	0.1152	0.5440	0.4810	6	0.8200	1.0914	1.0117	1	0.3181	1.29548	1.2216	3	0.3094
Item 7	0.0218	0.0220	1	0.8825	1.0281	1.0611	1	0.3066	0.5762	0.6444	6	0.6943	1.7045	1.8459	1	0.1788	1.00481	1.1758	3	0.3261
Item 8	0.6689	1.0237	1	0.3151	4.1053	7.0444	1	0.0099	0.8800	1.3264	6	0.2590	1.5758	2.4677	1	0.1208	0.56471	1.0201	3	0.3897
Item 9	0.3950	0.3947	1	0.5319	0.7810	0.5517	1	0.4602	1.1868	1.1186	6	0.3620	0.0061	0.0044	1	0.9473	2.15465	1.6054	3	0.1968
Item 10	0.1662	0.2226	1	0.6385	0.0562	0.0747	1	0.7854	0.4779	0.5930	6	0.7347	1.7091	2.2860	1	0.1351	0.23713	0.3081	3	0.8194
Item 11	2.2102	2.7641	1	0.1008	0.7307	0.7878	1	0.3778	0.7195	0.8213	6	0.5576	0.1186	0.1313	1	0.7182	1.38813	1.4720	3	0.2305
Item 12	0.8407	0.4672	1	0.4965	4.0381	2.5680	1	0.1136	1.3813	0.7263	6	0.6300	1.3914	0.7588	1	0.3867	1.77195	0.9691	3	0.4129
Item 13	0.0022	0.0032	1	0.9548	0.4744	0.7401	1	0.3926	0.2189	0.3072	6	0.9309	0.7383	1.0964	1	0.2988	0.92594	1.3496	3	0.2664
Item 14	0.8060	0.8604	1	0.3568	1.5836	1.6795	1	0.1993	0.1553	0.1493	6	0.9885	0.0001	0.0001	1	0.9924	1.10229	1.1866	3	0.3219
Item 15	0.0011	0.0013	1	0.9718	6.5196	8.4535	1	0.0049	0.2081	0.2695	6	0.9491	0.0905	0.1147	1	0.7359	0.48261	0.5642	3	0.6406
Item 16	0.0604	0.0620	1	0.8041	0.1303	0.1407	1	0.7087	1.4919	1.7747	6	0.1197	4.1714	4.7135	1	0.0335	0.19302	0.1957	3	0.8990
Item 17	0.0113	0.0118	1	0.9137	0.4919	0.5460	1	0.4625	0.6322	0.6574	6	0.6841	0.6411	0.6938	1	0.4078	0.91825	0.9483	3	0.4228
Item 18	0.0271	0.0240	1	0.8773	2.4678	2.5611	1	0.1141	0.2772	0.2291	6	0.9657	1.9227	1.7451	1	0.1909	4.29586	4.5724	3	0.0058
Item 19	0.1320	0.2377	1	0.6274	0.6241	1.1622	1	0.2848	0.4540	0.8276	6	0.5531	0.2442	0.4230	1	0.5176	0.37255	0.6246	3	0.6017
Item 20	0.0017	0.0015	1	0.9695	0.9790	0.8594	1	0.3571	0.7515	0.6323	6	0.7038	0.2903	0.2529	1	0.6166	1.60798	1.4700	3	0.2311
Item 21	0.2982	0.7187	1	0.3995	0.5608	1.2711	1	0.2635	0.2327	0.4860	6	0.8163	0.6207	1.2547	1	0.2666	0.90736	2.0239	3	0.1196
Item 22	1.8994	2.0254	1	0.1591	8.6295	10.2874	1	0.0020	0.6926	0.6871	6	0.6607	3.2329	3.9684	1	0.0503	1.94987	2.2745	3	0.0884
Item 23	1.6949	1.2076	1	0.2755	2.5257	1.8287	1	0.1807	1.7147	1.1836	6	0.3268	1.8298	1.4407	1	0.2342	1.32007	1.0292	3	0.3858
Item 24	1.7763	2.0754	1	0.1542	0.0384	0.0452	1	0.8323	0.9067	1.0678	6	0.3917	0.0405	0.0463	1	0.8303	0.18602	0.1944	3	0.8999
Item 25	1.5640	1.1936	1	0.2783	0.1401	0.1025	1	0.7498	1.2638	0.9177	6	0.4884	0.0138	0.0098	1	0.9215	1.11543	0.8427	3	0.4756
Item 26	2.2266	3.2015	1	0.0779	0.0324	0.0445	1	0.8335	0.3814	0.5010	6	0.8053	0.0302	0.0426	1	0.8371	0.80677	1.2067	3	0.3147
Item 27	0.1550	0.2799	1	0.5984	1.0708	2.0280	1	0.1589	0.3142	0.5510	6	0.7674	0.3447	0.6437	1	0.4251	0.82152	1.5163	3	0.2188
Item 28	0.0017	0.0025	1	0.9604	3.7263	5.9342	1	0.0174	0.5205	0.7847	6	0.5852	0.4097	0.5882	1	0.4458	0.58555	0.9055	3	0.4435
Item 29	0.0212	0.0541	1	0.8167	0.7854	2.0109	1	0.1606	0.5694	1.7091	6	0.1335	0.0717	0.1877	1	0.6662	0.4659	1.1719	3	0.3274

Revision and validation of the German version of the Systemic Sclerosis Quality of Life Questionnaire (SScQoL) using Rasch analysis; Orphanet Journal of Rare Disease; Kocher, A., Ndosi, N., Denhaerynck, K., Simon, M., Dwyer A.A., Distler, O., Hoeper, K., Künzler-Heule, P., Redmond, A.C., Villiger, P.M., Walker, U.A., Nicca, D.; Institute of Nursing Science (INS), Department Public Health (DPH), Faculty of Medicine, University of Basel, Switzerland, dunja.nicca@unibas.ch
