# Supplementary material for: A rare disease patient-reported outcome measure: revision and validation of the German version of the Systemic Sclerosis Quality of Life Questionnaire (SScQoL) using the Rasch model
Source: Orphanet J Rare Dis. 2021 Aug 9;16:356. doi: 10.1186/s13023-021-01944-9 (PMC8351336; doi:10.1186/s13023-021-01944-9)
Supplement: Supplementary file 5 — Additional File 5. Differential item functioning (DIF) analysis B. [file 13023_2021_1944_MOESM5_ESM.pdf]

Additional file 5 Differential item functioning (DIF) analysis B

	DIF by Sex (M vs F) = Not significant				DIF by Age (62 vs 63+) = Not significant				DIF by education level (7 levels) = Not significant				DIF by disease duration (9 vs 10+) = Not significant				DIF by disease subgroup (lSSc/dSSc/uSSc/Unknown) = Not significant			
**Domain**	**MS**	**F**	**DF**	**p-value***	**MS**	**F**	**DF**	**p-value***	**MS**	**F**	**DF**	**p-value***	**MS**	**F**	**DF**	**p-value***	**MS**	**F**	**DF**	**p-value***
Function	0.19281	0.21226	1	0.6465	4.80378	5.37441	1	0.0235	0.564	0.65788	6	0.6837	0.99794	1.06801	1	0.305112	0.51055	0.57742	3	0.632022
Emotional	0.16277	0.26736	1	0.6068	1.22602	2.0194	1	0.1601	0.7317	1.22829	6	0.3055	1.08488	1.8127	1	0.182934	0.14922	0.23207	3	0.873683
Sleep	0.09851	0.08185	1	0.7756	0.17544	0.14115	1	0.7083	0.82312	0.65447	6	0.6864	0.19451	0.15662	1	0.693511	1.86525	1.70591	3	0.174649
Social	0.02278	0.03089	1	0.8610	0.05905	0.08235	1	0.7750	0.13609	0.17504	6	0.9826	0.39224	0.53712	1	0.466264	0.34019	0.43505	3	0.728702
Pain	0.25705	0.39217	1	0.5332	0.74814	1.13911	1	0.2897	0.39402	0.57958	6	0.7451	0.32009	0.48379	1	0.489116	0.83444	1.34318	3	0.268556

DIF, Differential Item Functioning; MS, Mean Square; F, F-Value; DF, degree of freedom; p-value*, Bonferroni adjusted p-value i.e. 0.05/number of tests (items)

Revision and validation of the German version of the Systemic Sclerosis Quality of Life Questionnaire (SScQoL) using Rasch analysis; Orphanet Journal of Rare Diseases; Kocher, A., Ndosi, N., Denhaerynck, K., Simon, M., Dwyer A.A., Distler, O., Hoeper, K., Künzler-Heule, P., Redmond, A.C., Villiger, P.M., Walker, U.A., Nicca, D.; Institute of Nursing Science (INS), Department Public Health (DPH), Faculty of Medicine, University of Basel, Switzerland, dunja.nicca@unibas.ch
