# Supplementary material for: A rare disease patient-reported outcome measure: revision and validation of the German version of the Systemic Sclerosis Quality of Life Questionnaire (SScQoL) using the Rasch model
Source: Orphanet J Rare Dis. 2021 Aug 9;16:356. doi: 10.1186/s13023-021-01944-9 (PMC8351336; doi:10.1186/s13023-021-01944-9)
Supplement: Supplementary file 6 — Additional File 6. Testing the dichotomized responses. [file 13023_2021_1944_MOESM6_ESM.pdf]

Additional file 6 Testing the dichotomized responses

(i.e. 'always', 'usually' or 'sometimes'=1, 'never'=0)

Additional file 6A: fit statistics for individual items with dichotomised response structure

Item	Location	SE	FitResid	DF	ChiSq	DF	p-value*
Item 1	0.363	0.315	0.709	68.5	1.806	1	0.178969
Item 2	2.18	0.4	-0.432	68.5	0.723	1	0.395106
Item 3	-0.097	0.311	-0.249	66.6	0.099	1	0.753411
Item 4	-0.004	0.311	0.474	67.55	1.362	1	0.243172
Item 5	1.475	0.353	0.57	68.5	1.069	1	0.301237
Item 6	-0.021	0.312	0.369	66.6	0.318	1	0.57284
Item 7	0.236	0.313	0.161	67.55	1.279	1	0.258149
Item 8	-0.019	0.312	-1.245	67.55	0.676	1	0.411119
Item 9	-0.028	0.312	-1.215	67.55	0.676	1	0.410891
Item 10	-0.134	0.311	0.132	67.55	0.152	1	0.696616
Item 11	0.186	0.312	-0.9	68.5	1.325	1	0.249663
Item 12	2.569	0.438	0.049	68.5	1.858	1	0.172868
Item 13	0.06	0.314	-1.806	66.6	1.604	1	0.205356
Item 14	1.628	0.367	-0.465	67.55	0.117	1	0.732811
Item 15	0.81	0.331	-1.03	66.6	0.73	1	0.392728
Item 16	-0.329	0.31	0.182	65.65	1.2	1	0.273324
Item 17	0.054	0.314	-1.632	66.6	0.355	1	0.551372
Item 18	1.681	0.37	-0.742	67.55	1.216	1	0.270162
Item 19	-2.29	0.331	-0.989	67.55	4.809	1	0.028316
Item 20	-0.329	0.309	-0.008	67.55	0.052	1	0.819643
Item 21	-2.35	0.335	-1.304	66.6	6.517	1	0.010687
Item 22	0.969	0.335	-0.156	67.55	1.22	1	0.269341
Item 23	1.206	0.34	0.557	67.55	0.564	1	0.452666
Item 24	-2.67	0.346	0.037	66.6	2.775	1	0.095764
Item 25	-2.521	0.342	0.687	67.55	11.357	1	0.000753
Item 26	0.976	0.333	-0.957	67.55	0.756	1	0.384706
Item 27	-1.759	0.32	-1.141	68.5	4.987	1	0.025535
Item 28	-0.846	0.311	-0.007	67.55	0.863	1	0.352842
Item 29	-0.992	0.31	-1.29	68.5	1.734	1	0.187852

Additional file 6B: fit statistics for domains with dichotomised response structure

Domain	Location	SE	FitResid	DF	ChiSq	DF	p-value*
Function	0.618	0.129	0.215	51.65	1.339	1	0.247277
Emotional	-0.075	0.079	-1.05	49.4	0.202	1	0.652785
Sleep	-0.294	0.172	0.236	53.15	0.038	1	0.844455
Social	-0.16	0.109	-0.22	50.15	1.933	1	0.164422
Pain	-0.089	0.19	-0.13	51.65	1.757	1	0.184966

Revision and validation of the German version of the Systemic Sclerosis Quality of Life Questionnaire (SScQoL) using Rasch analysis; Orphanet Journal of Rare Diseases; Kocher, A., Ndosi, N., Denhaerynck, K., Simon, M., Dwyer A.A., Distler, O., Hoeper, K., Künzler-Heule, P., Redmond, A.C., Villiger, P.M., Walker, U.A., Nicca, D.; Institute of Nursing Science (INS), Department Public Health (DPH), Faculty of Medicine, University of Basel, Switzerland, dunja.nicca@unibas.ch
