# Supplementary material for: A rare disease patient-reported outcome measure: revision and validation of the German version of the Systemic Sclerosis Quality of Life Questionnaire (SScQoL) using the Rasch model
Source: Orphanet J Rare Dis. 2021 Aug 9;16:356. doi: 10.1186/s13023-021-01944-9 (PMC8351336; doi:10.1186/s13023-021-01944-9)
Supplement: Supplementary file 7 — Additional File 7. Scoring instructions for the new German version of the Systemic Sclerosis Quality of Life (SScQoL) questionnaire. [file 13023_2021_1944_MOESM7_ESM.pdf]

Additional file 7 Scoring instructions for the new German version of the Systemic Sclerosis Quality of Life (SScQoL) questionnaire

German SSc-QoL – Scoring instructions

How to use and score the German Systemic Sclerosis Quality of Life (SScQoL) tool

Background information

The German SScQoL is a self-administered questionnaire containing 29 items, which measure health-related quality of life of patients with systemic sclerosis (SSc). The statements are grouped into five domains that measure specific aspects of quality of life according to the International Classification of Functioning, Disability and Health (ICF) framework. These are:

1. Function
2. Emotional
3. Sleep
4. Social
5. Pain

Uses of the German SScQoL

The German SScQoL can be used by clinicians in wards or clinics to assess quality of life of individual SSc patients. The SScQoL can also be used to assess quality of life of groups of SSc patients. Here should be considered that the German SScQoL is only validated in Swiss German-speaking patients. Thus, caution is needed when attempting to extend findings to other German-speaking populations. Further studies would need to include patients from Austria, Germany and Liechtenstein to confirm the robustness of the German SScQoL.

How is the German SScQoL completed?

The German SScQoL is designed to be completed by the patient unaided. The 29 statements (items) comprise 4-point response options with the following descriptives: “Always”, “Usually”, “Sometimes”, and “Never”. Patients should tick “X” in the box that corresponds with their level of agreement. Only one box should be ticked for each statement.

How the German SScQoL is scored?

The German SScQoL can be used as a tool in clinical practice to evaluate individual patient quality of life or as a research/audit tool to evaluate health related quality of life of groups of patients. Two ways of scores can be used as shown below.

German SSc-QoL – Scoring instructions

A. The German SScQoL used as a clinical tool

The clinician may want to know how health-related quality of life of a SSc patient is at a particular time, which allows her/him to focus on specific items or overall quality of life during the consultation. Furthermore, quality of life can be assessed again at a follow-up consultation to see changes over time. Therefore, the German SScQoL can be scored as follows:

1. Ich kann nichts tun, ohne vorher gründlich drüber nachzudenken

   3 Immer 2 Meistens 1 Manchmal 0 Nie

2. Die Erkrankung ist ständig in meinen Gedanken

   3 Immer 2 Meistens 1 Manchmal 0 Nie

3. Ich mache mir Sorgen, dass ich andere Menschen im Stich lasse

   3 Immer 2 Meistens 1 Manchmal 0 Nie

4. Mein derzeitiger Gesundheitszustand macht mich wütend

   3 Immer 2 Meistens 1 Manchmal 0 Nie

5. Ich rege mich auf, wenn ich etwas nicht mehr tun kann

   3 Immer 2 Meistens 1 Manchmal 0 Nie

6. Ich bin oft frustriert

   3 Immer 2 Meistens 1 Manchmal 0 Nie

7. Ich kann mich nicht darauf verlassen, wie es mir am nächsten Tag gehen wird

   3 Immer 2 Meistens 1 Manchmal 0 Nie

8. Ich fühle mich, als ob ich ständig kämpfen würde

   3 Immer 2 Meistens 1 Manchmal 0 Nie

9. Durch meine Erkrankung habe ich Schlafstörungen

   3 Immer 2 Meistens 1 Manchmal 0 Nie

Subtotal Seite 1_________

German SSc-QoL – Scoring instructions

10. Die Erkrankung beeinträchtigt mein Sozialleben sehr

3 Immer 2 Meistens 1 Manchmal 0 Nie

11. Die Erkrankung hat Einfluss auf das Befinden der Menschen in meinem Umfeld

3 Immer 2 Meistens 1 Manchmal 0 Nie

12. Meine Hände funktionieren nicht mehr so gut wie früher

3 Immer 2 Meistens 1 Manchmal 0 Nie

13. Die Erkrankung belastet meine persönlichen Beziehungen

3 Immer 2 Meistens 1 Manchmal 0 Nie

14. Ich muss mich häufiger ausruhen

3 Immer 2 Meistens 1 Manchmal 0 Nie

15. Jede Art von Tätigkeit ist mit Schwierigkeiten verbunden

3 Immer 2 Meistens 1 Manchmal 0 Nie

16. Ich vermeide gewisse gesellschaftliche Situationen, um mich nicht in Verlegenheit zu bringen

3 Immer 2 Meistens 1 Manchmal 0 Nie

17. Ich nehme mir Dinge zu Herzen, die mich früher nicht bedrückt hätten

3 Immer 2 Meistens 1 Manchmal 0 Nie

18. Das Leben ist einfach nicht mehr wie früher

3 Immer 2 Meistens 1 Manchmal 0 Nie

19. Ich komme überhaupt nicht zurecht

3 Immer 2 Meistens 1 Manchmal 0 Nie

20. Schlecht zu schlafen beeinträchtigt mich sehr

3 Immer 2 Meistens 1 Manchmal 0 Nie

21. Ich fühle mich sehr isoliert

3 Immer 2 Meistens 1 Manchmal 0 Nie

_______ **Subtotal Seite 2**

German SSc-QoL – Scoring instructions

22. Hausarbeiten können ein Problem sein

3 Immer 2 Meistens 1 Manchmal 0 Nie

23. Ich musste einige meiner Hobbies aufgeben

3 Immer 2 Meistens 1 Manchmal 0 Nie

24. Ich fühle mich schuldig, krank zu sein

3 Immer 2 Meistens 1 Manchmal 0 Nie

25. Ich habe Schwierigkeiten mich selbst so zu waschen, wie ich gerne möchte

3 Immer 2 Meistens 1 Manchmal 0 Nie

26. Die Schmerzen schränken mich in meinem Handeln ein

3 Immer 2 Meistens 1 Manchmal 0 Nie

27. Ich fühle mich hilflos

3 Immer 2 Meistens 1 Manchmal 0 Nie

28. Die Schmerzen laugen mich aus

3 Immer 2 Meistens 1 Manchmal 0 Nie

29. Ich vermisse es, meine Angelegenheiten selbst erledigen zu können

3 Immer 2 Meistens 1 Manchmal 0 Nie

_______ **Subtotal Seite 3**

_______ **Subtotal Seite 2**

_______ **Subtotal Seite 1**

_______ **TOTAL (von insgesamt 87 Punkten)**

German SSc-QoL – Scoring instructions

B. The German SScQoL used as a survey or research tool (scored dichotomously)

For use in audit or research, the German SScQoL needs to be coded and scored using the following steps:

a) The German SScQoL scales descriptives should be number-coded thus:

Immer	= 1
Meistens	= 1
Manchmal	= 1
Nie	= 0

See example below:

Ich habe Schwierigkeiten mich selbst so zu waschen, wie ich gerne möchte

1	Immer	1	Meistens	1	Manchmal	0	Nie

Die Schmerzen schränken mich in meinem Handeln ein

1	Immer	1	Meistens	1	Manchmal	0	Nie

b) Following the coding, adding up all the item scores gives the total German SScQoL score (range = 0–29).

Important remark:

Comparisons with data collected with other SScQoL language versions should be avoided unless further cross-cultural validity tests are done to assess measurement equivalence of the German SScQoL with other language versions and thus enable multinational comparisons and data pooling.

The SScQoL should be cited using the following validation studies:

1. Ndosi M, Alcacer-Pitarch B, Allanore Y, et al. Common measure of quality of life for people with systemic sclerosis across seven European countries: a cross-sectional study. Annals of the Rheumatic Diseases 2018;77:1032-1038.
2. Kocher A, Ndosi M, Hoeper K, et al THU0623-HPR revision and validation of the German version of the systemic sclerosis quality of life questionnaire (SScQoL) with Mokken scale analysis. Annals of the Rheumatic Diseases 2020;79:554-555
